# Supplementary material for: Invasive lobular carcinoma: integrated multi-omics analysis reveals silencing of Argininosuccinate synthase and upregulation of nucleotide biosynthesis in tamoxifen resistance
Source: Cell Death Dis. 2025 Jul 11;16(1):514. doi: 10.1038/s41419-025-07788-6 (PMC12254388; doi:10.1038/s41419-025-07788-6)
Supplement: Supplementary file 1 — Supplementary Material [file 41419_2025_7788_MOESM1_ESM.docx]

**Supplementary Fig. S1**


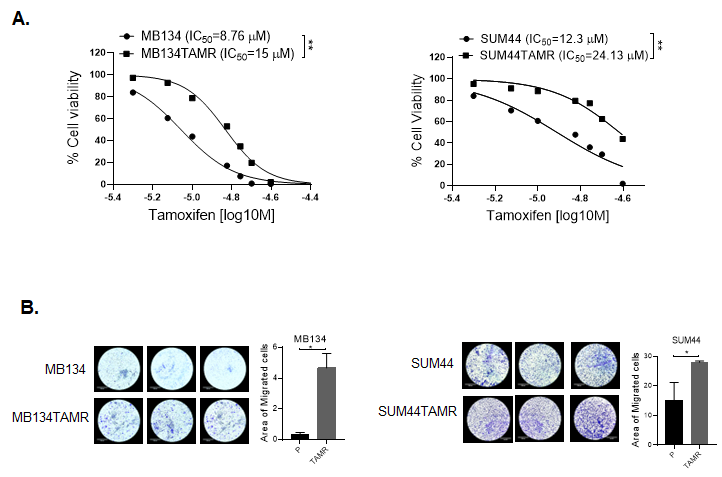


**Supplementary Fig. S1**: **Increase in tamoxifen tolerance and migratory property of TAMR–ILC cell lines. A.** MB134 vs. MB134-TAMR (left panel) and SUM44 vs. SUM44-TAMR (right panel) cells were treated with 0–25 µM TAM for 5 days. Change in cell viability was determined by manual counting of cells every 24 hours. IC_50_ for TAM was determined by plotting the data using GraphPad Prism10 software. **B.** Transwell Boyden chamber assays assessing the migratory capacity of parental and tamoxifen-resistant (TAMR) ILC cell lines conducted over 72 hours after cell seeding. The area covered by migrated cells was quantified and presented as a bar graph. Data represent mean ± SEM from at least three independent experiments. MB134 vs. MB134-TAMR (left panel) and SUM44 vs. SUM44-TAMR (right panel).

**Supplementary Fig. S2**


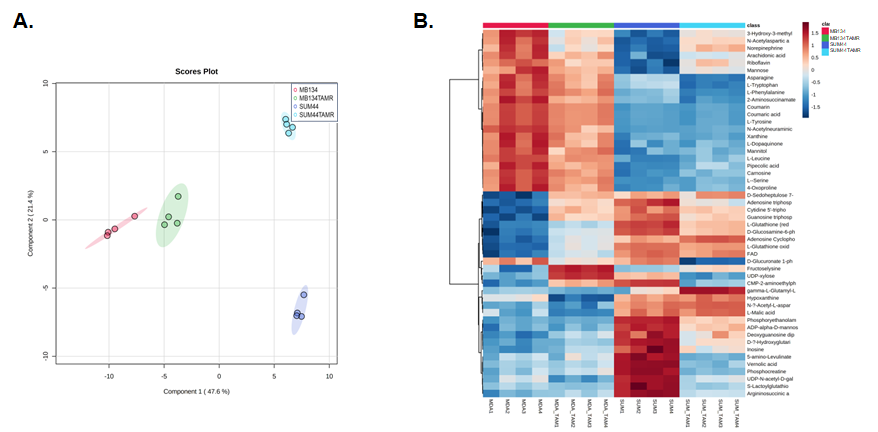


**Supplementary Fig. S2**: Mutually deregulated metabolic pathways between parental and TAMR ILC cell lines. **A.** Partial Least Squares Discriminant Analysis (PLS-DA) comparing the overall metabolic profiles of the parental and TAMR ILC cell lines, illustrating the separation of metabolic profiles between the four cell lines. **B.** Heatmap depicting the relative abundance of each metabolite across the parental and TAMR ILC cell lines. Each row represents a metabolite, while each column represents a cell line. The color intensity corresponds to the abundance level of each metabolite, with red indicating higher abundance and blue indicating lower abundance (scale shown). The heatmap highlights the alterations in metabolic pathways that are deregulated between the parental and tamoxifen resistant ILC cell lines.

**Supplementary Fig. S3**


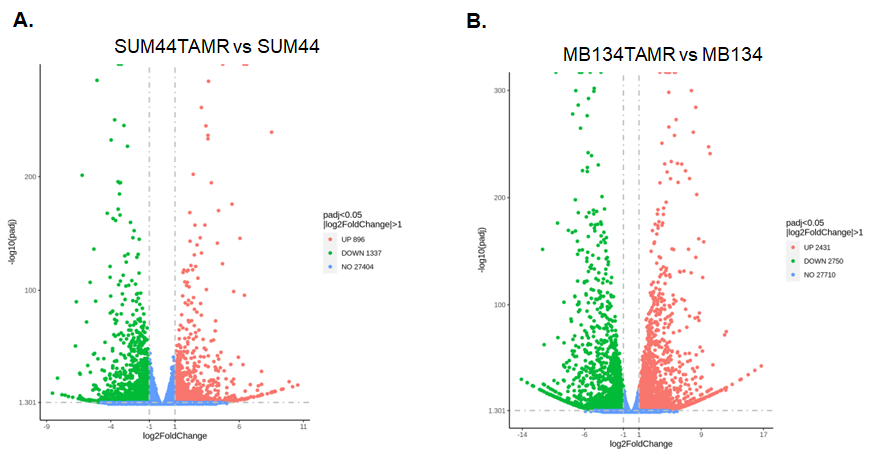


**Supplementary Fig S3:** RNA seq analysis. **A.** Volcano plot of RNA sequencing data showing differential expression of genes and their statistical significance in SUM44 vs. SUM44TAMR, and **B.** MB134 vs. MB134TAMR cell lines.


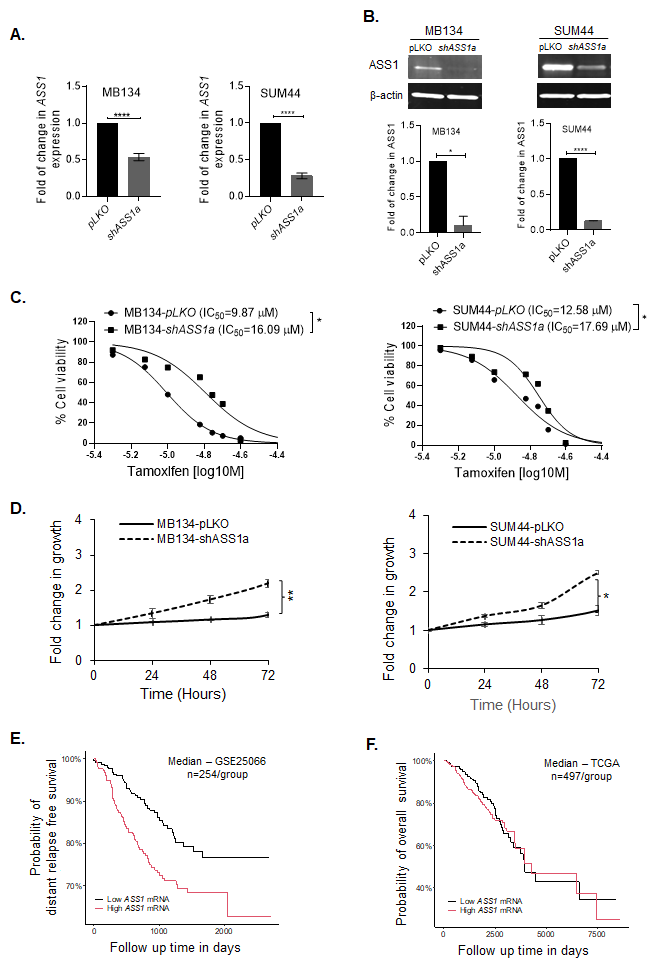


**Supplementary Fig. S4**

**Supplementary Fig. S4: Knockdown of *ASS1* in ILC cell lines using shRNA.** **A.** Expression and **B.** protein level analysis of ASS1 in MB134-*shASS1a* and SUM44- *shASS1a* cells compared to the control cells (pLKO), quantified in the bar diagram. **C.** Dose response to tamoxifen in MB134-pLKO and MB134-*shASS1a* cells (left panel) and SUM44-pLKO and SUM44*-shASS1a* cells (right panel). Overnight cultures of exponentially growing cells were treated with vehicle or drugs for 5 days. IC_50_ for TAM was calculated using GraphPad Prism 10. **D.** Growth kinetics of MB134-pLKO and MB134-*shASS1a* cells (left panel) and SUM44-pLKO and SUM44*-shASS1a* cells (right panel). Fold change in growth normalized to day 0 at each time point over 72 hours. **E.** Distant relapse free survival analysis of all breast cancer patients in relation to *ASS1* expression using Hatzis data set (GSE25006)^1^. **F.** Overall survival analysis of all breast cancer patients in relation to *ASS1* expression using TCGA data set^2^.

^1^ A genomic predictor of response and survival following taxane-anthracycline chemotherapy for invasive breast cancer. Hatzis .et. al. JAMA. 305(18):1873-81. *(doi: 10.1001/jama.2011.593.)*

^2^ <https://www.cbioportal.org>

**Supplementary Fig. S5**


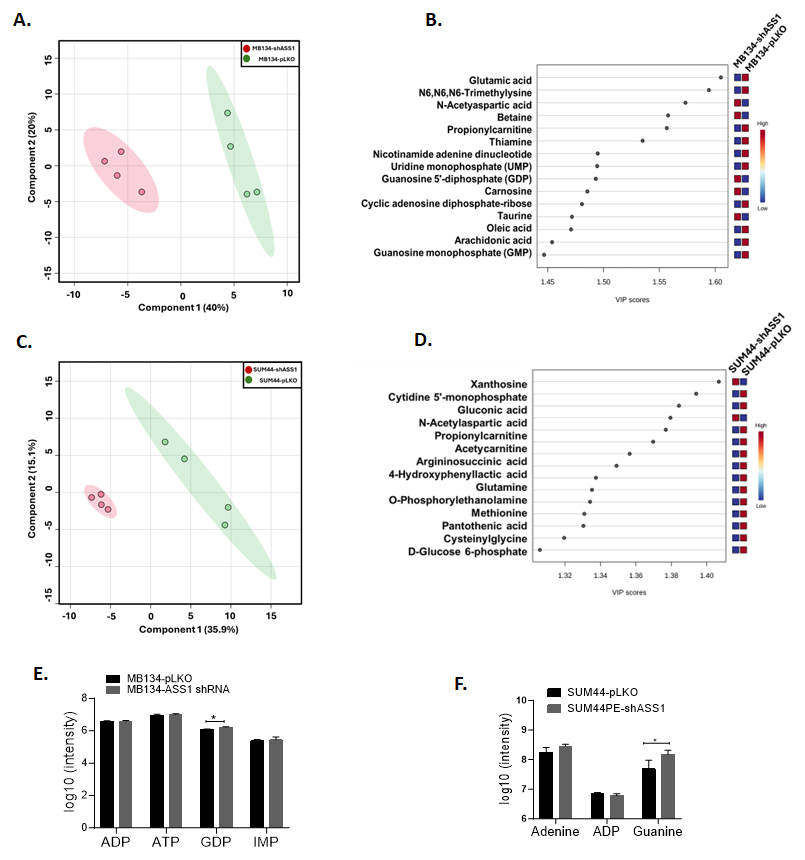


**Supplementary Fig S5: A.** Partial Least Squares Discriminant Analysis (PLS-DA) plot comparing metabolic profile of MB134-*shASS1* vs. MB134-pLKO cells. **B.** Variance Importance in Projection (VIP) plot highlighting the top metabolites driving the separation of the MB134-*shASS1* vs. MB134-pLKO cells. **C.** PLS-DA plot comparing metabolic profile of SUM44-*shASS1* vs. SUM44-pLKO cells. **D.** VIP plot highlighting the top metabolites driving the separation of the SUM44-*shASS1* vs. SUM44-pLKO cells.

**Supplementary Fig. S6**


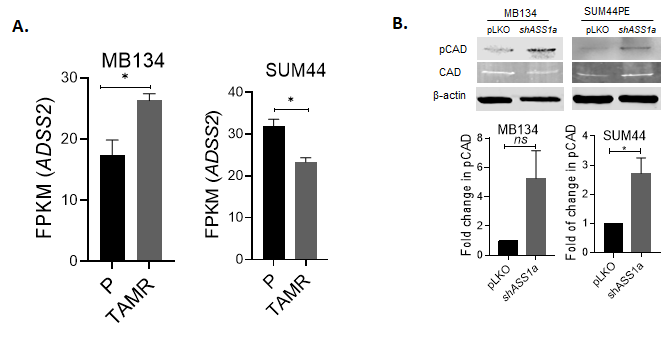


**Supplementary Fig S6.** **A.** Expression analysis of *ADSS2* in MB134TAMR vs. MB134 (left panel) and SUM44TAMR vs. SUM44 (right panel) cell lines using Fragments Per Kilobase of transcript per Million mapped reads (FPKM) from RNA sequencing data. P- Parental, TAMR-Tamoxifen resistant. **B.** Representative picture of pCAD^S1859^ in MB134-pLKO and MB134-*shASS1a* cells (left panel) and SUM44-pLKO and SUM44*-shASS1a* cells (right panel). Bar diagrams show average of more than one independent experiments (n=2). *p <0.05.

**Supplemental Table 1:** A full list of the significantly deregulated pathways between the queried cell pairs.

**
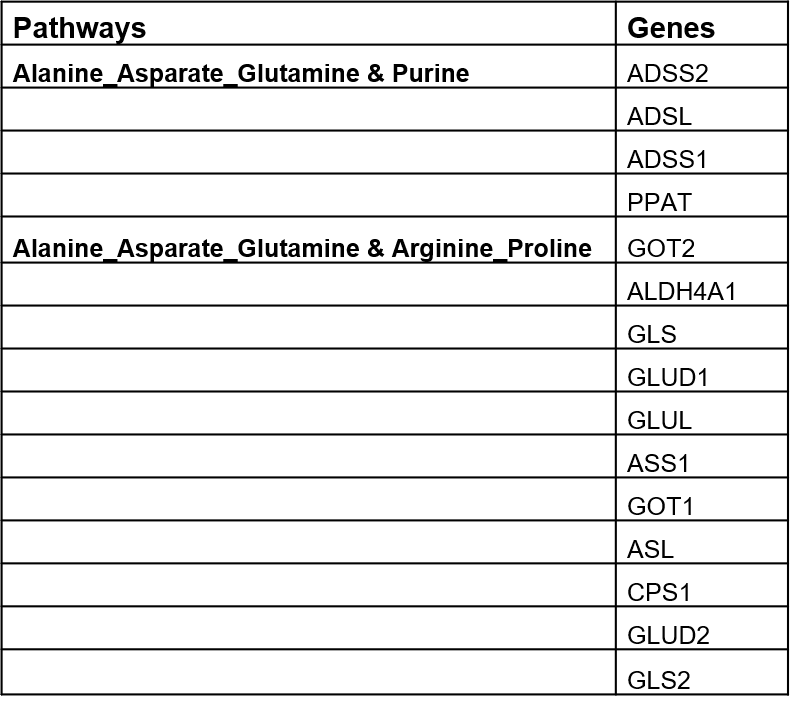
**

**Supplementary Table 2:** List of genes involved in the three dysregulated pathways
