# Supplementary material for: Invasive lobular carcinoma: integrated multi-omics analysis reveals silencing of Argininosuccinate synthase and upregulation of nucleotide biosynthesis in tamoxifen resistance
Source: Cell Death Dis. 2025 Jul 11;16(1):514. doi: 10.1038/s41419-025-07788-6 (PMC12254388; doi:10.1038/s41419-025-07788-6)
Supplement: Supplementary file 2 — Supplementary file: Full western blot images [file 41419_2025_7788_MOESM2_ESM.pptx]

## Slide 1
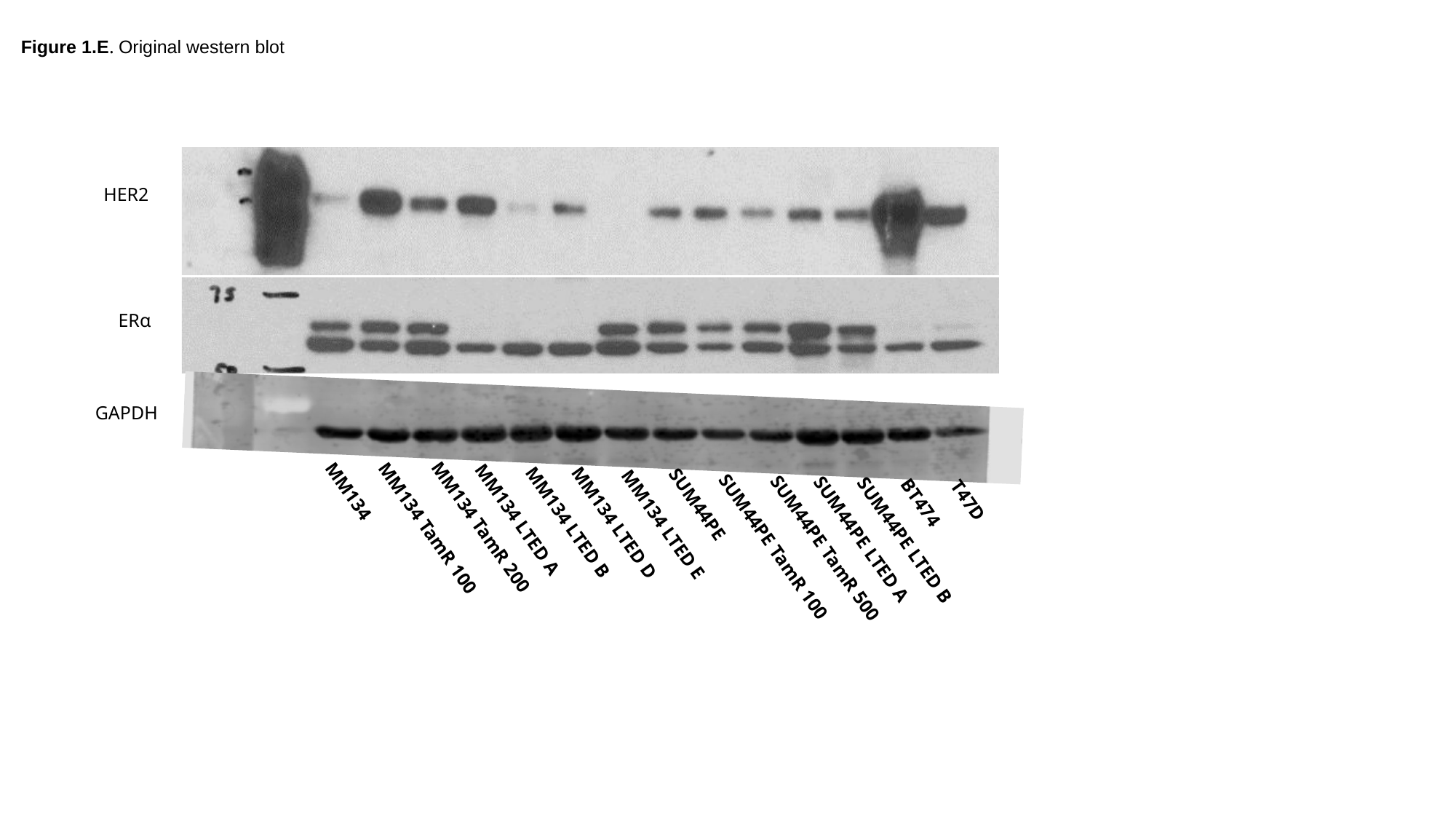

Figure 1.E. Original western blot
HER2
ERα
GAPDH
MM134 TamR 200
MM134
MM134 TamR 100
MM134 LTED A
MM134 LTED D
MM134 LTED B
SUM44PE
MM134 LTED E
SUM44PE TamR 100
SUM44PE TamR 500
SUM44PE LTED A
SUM44PE LTED B
BT474
T47D

## Slide 2
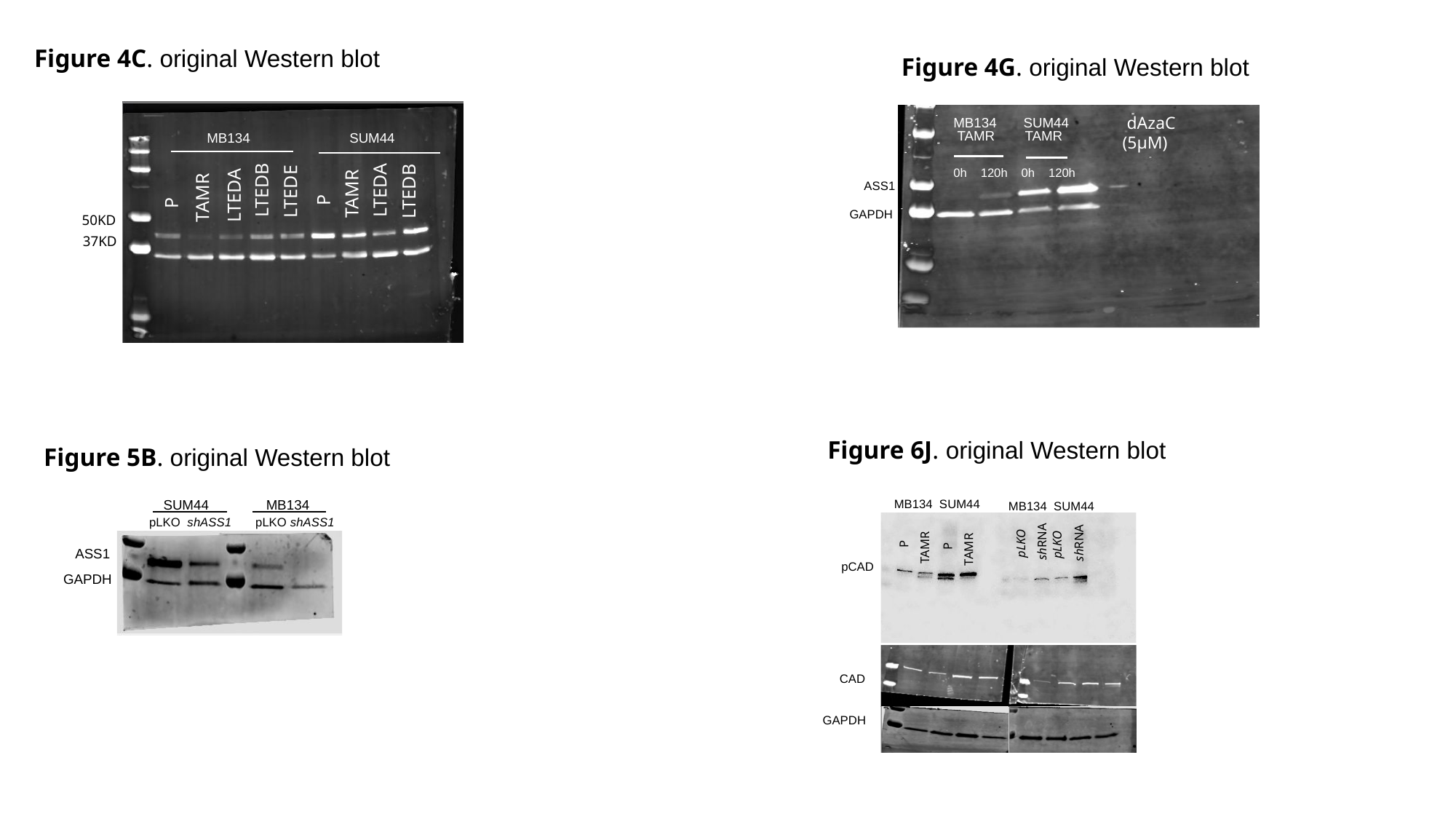

Figure 4C. original Western blot
Figure 4G. original Western blot
MB134 SUM44
LTEDA
LTEDE
LTEDB
TAMR
 LTEDB
LTEDA
TAMR
P
P
134-LTED-E
134-LTED-D
SUM-TAMR
134-LTED-A
SUM-LTED-B
SUM-LTED-A
134-P
134-TAMR
SUM-P
50KD
37KD
 dAzaC (5µM)
MB134 SUM44
 TAMR TAMR
0h 120h 0h 120h
ASS1
GAPDH
Figure 6J. original Western blot
Figure 5B. original Western blot
SUM44 MB134
 pLKO shASS1 pLKO shASS1
ASS1
GAPDH
MB134 SUM44
MB134 SUM44
shRNA
shRNA
pLKO
P
pLKO
P
TAMR
TAMR
pCAD
CAD
GAPDH

## Slide 3
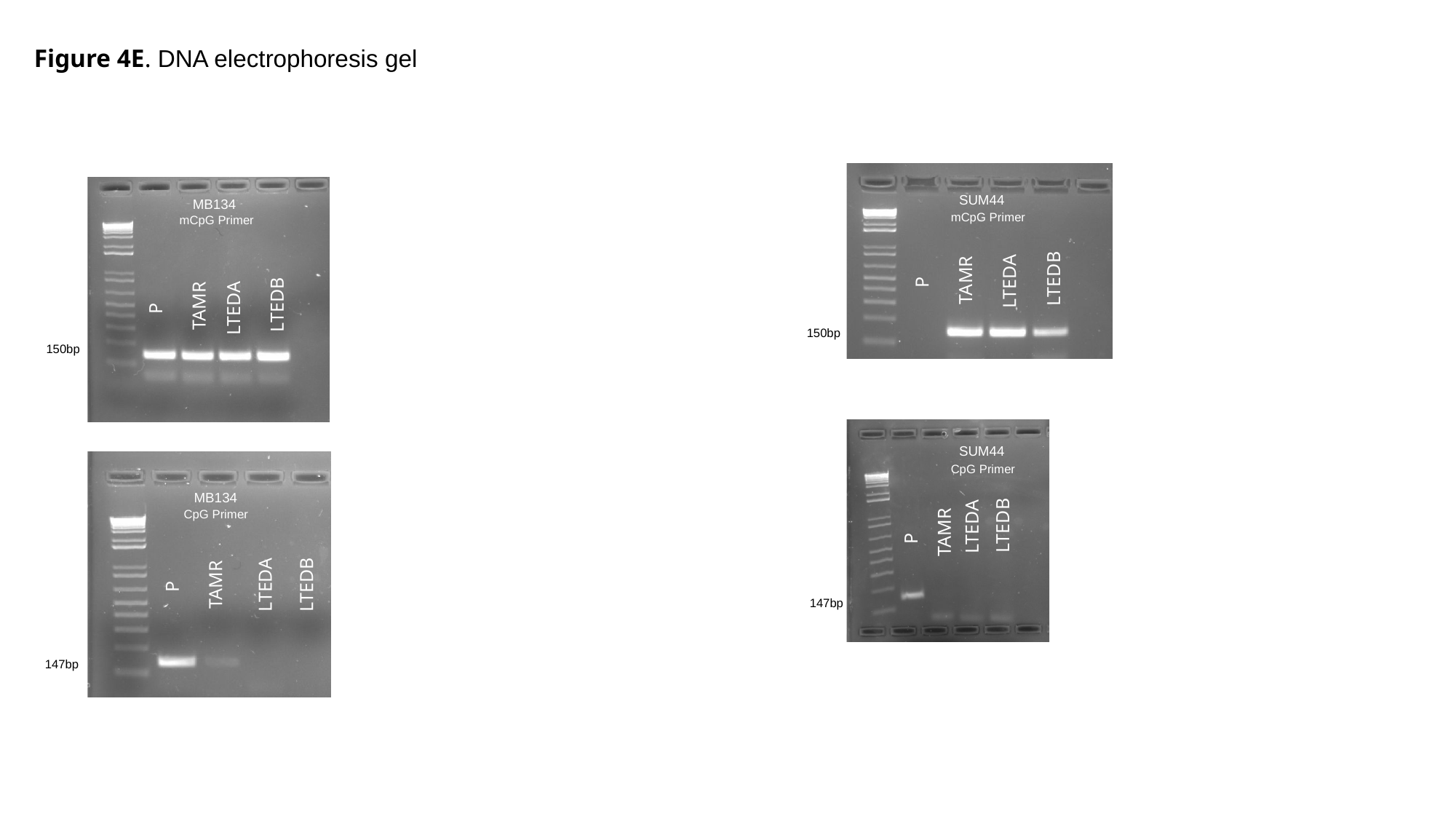

Figure 4E. DNA electrophoresis gel
SUM44
MB134
mCpG Primer
mCpG Primer
TAMR
LTEDA
P
 LTEDB
TAMR
P
LTEDA
 LTEDB
150bp
150bp
SUM44
CpG Primer
MB134
CpG Primer
LTEDA
 LTEDB
TAMR
P
LTEDA
TAMR
P
 LTEDB
147bp
147bp

## Slide 4
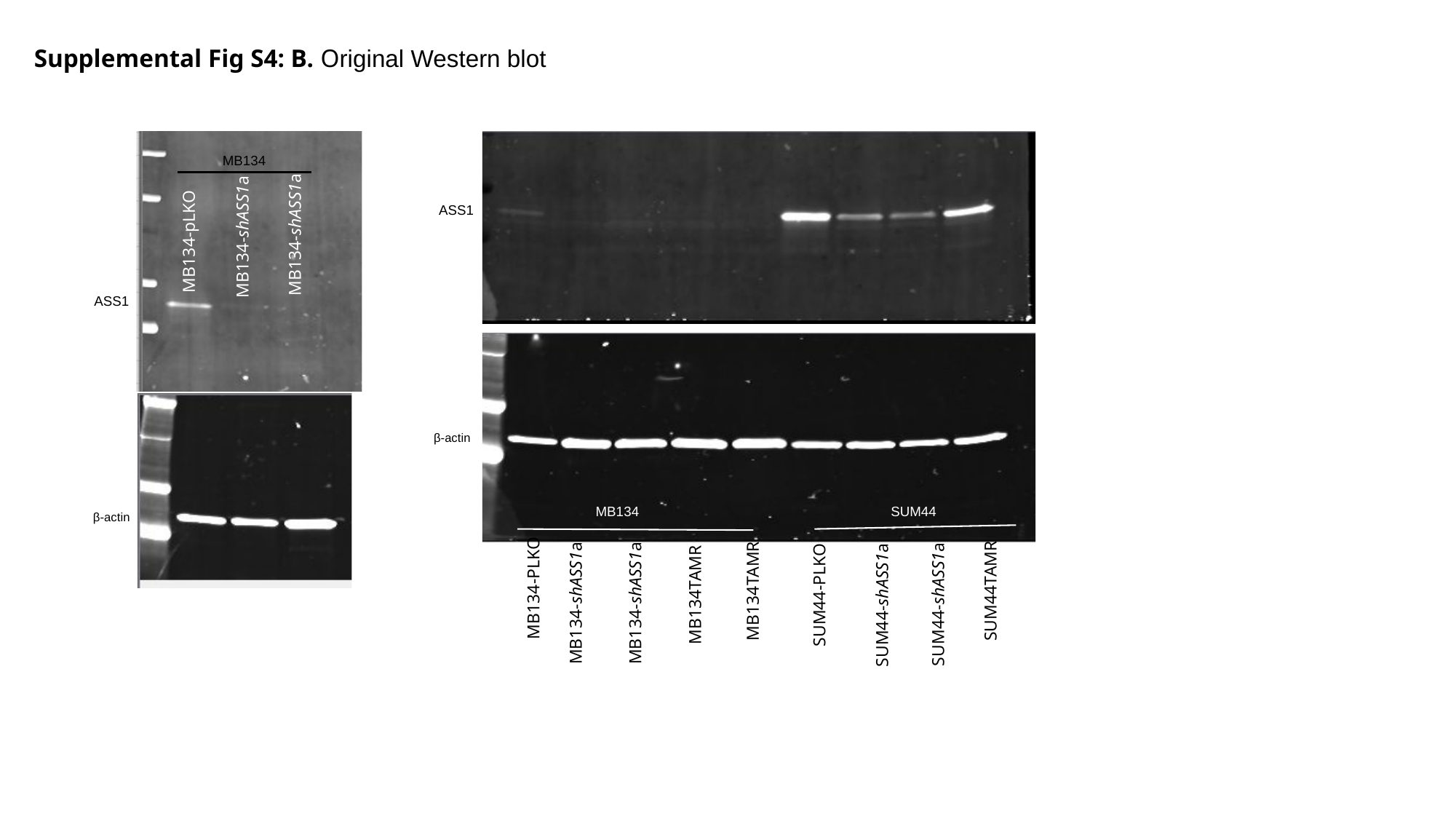

Supplemental Fig S4: B. Original Western blot
 MB134
MB134-shASS1a
MB134-pLKO
ASS1
MB134-shASS1a
ASS1
β-actin
 MB134 SUM44
β-actin
MB134-PLKO
MB134TAMR
SUM44TAMR
SUM44-PLKO
SUM44-shASS1a
SUM44-shASS1a
MB134TAMR
MB134-shASS1a
MB134-shASS1a

## Slide 5
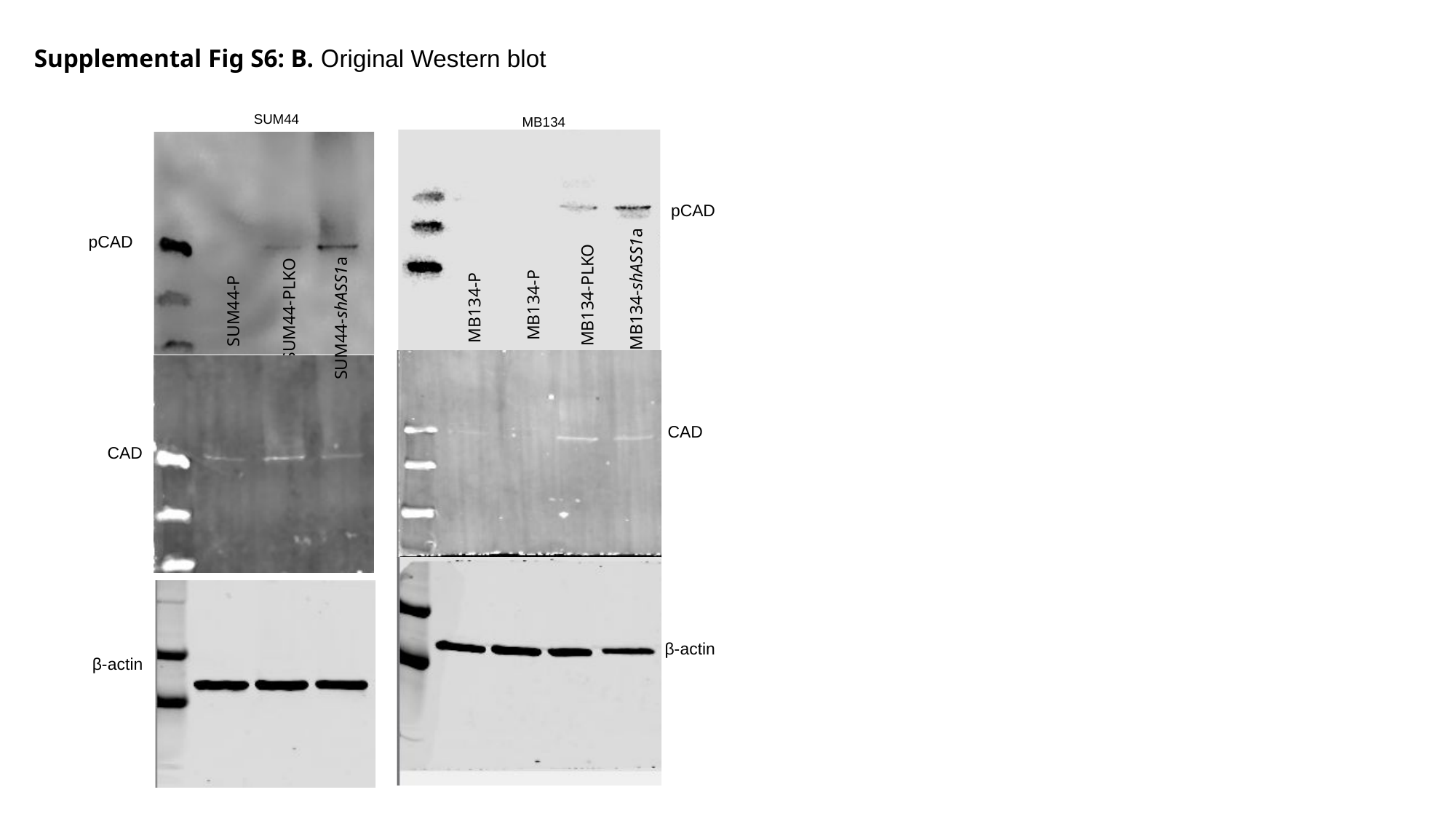

Supplemental Fig S6: B. Original Western blot
SUM44
MB134
pCAD
pCAD
MB134-PLKO
MB134-P
MB134-P
SUM44-PLKO
SUM44-P
MB134-shASS1a
SUM44-shASS1a
CAD
CAD
β-actin
β-actin
